# Bio-Inspired Energy-Efficient Nanofabricated Electrical Contacts

**DOI:** 10.3390/biomimetics11030211

**Published:** 2026-03-16

**Authors:** Ella M. Gale, Ilyas A. H. Farhat, Suha S. Azhar, Hanno Hildmann, Aaron Stein, A. F. Isakovic

**Affiliations:** 1 School of Chemistry, University of Bristol, Bristol BS8 1TS, UK; ella.gale@bristol.ac.uk; 2 Department of Electrical and Computer Engineering, University of Toronto, Toronto, ON M5S 3G4, Canada; ilyas.farhat@utoronto.ca; 3 Department of Mechanical Engineering, Khalifa University, Abu Dhabi P.O. Box 127788, United Arab Emirates; iregx137@gmail.com; 4 Netherlands Organisation for Applied Scientific Research, 2597 Den Haag, The Netherlands; hanno.hildmann@tno.nl; 5 Smart Sensor Systems Research Group, Department of Mechatronics, The Hague University of Applied Sciences, Rotterdamseweg 137, 2628 Delft, The Netherlands; 6 Center for Functional Nanomaterials, Brookhaven National Laboratory, Upton, NY 11973, USA; stein@bnl.gov; 7 Department of Physics, Illinois Wesleyan University, 1312 Park St., Bloomington, IL 61701, USA

**Keywords:** insect setae, bio-inspired contacts, NiSi (nickel silicide), nanocontacts energy efficiency, contact S/V ratio, contact geometry and shape, transport modes, charge scattering, I–V curves, noise

## Abstract

Nanoscale electrical contacts, especially those between materials of dissimilar electronic properties, often represent one of the main causes of drops in energy transfer efficiency. They are also among the sources of above-threshold noise, and their performance often decreases over the lifetime of the nanodevices. Scale-down limitations from mesoscopic to nanoscale devices, and likewise, of nanoscale to quantum-scale devices are also impeded by contacts’ quality. Making more reliable, energy-efficient electrical contacts is among the goals of the nanoelectronics research within the framework of energy-efficient electronic systems. This report focuses on the design, nanofabrication, and testing of novel shapes of electrical contacts. Lithography and nanofabrication were utilized to mimic the approximate shape of insect setae for mesoscale contacts design. The contacts are tested for elementary charge transport via I–V curves and for the broadband, 1/*f* noise. Tests show that contacts design leads to a measurable decrease in the energy necessary to operate a contact as a switch by at least 12–20%, depending on temperature, while broadband noise shows measurably lower power spectra, for bio-inspired contacts. The proposed method is open to modifications and improvements as required by various on-chip applications.

## 1. Introduction and Motivation

The causes of energy losses on a chip are complex and vary with the design and the purpose of the chip, but, one of the common causes is the issue of the efficiency of energy (power) transfer at the contacts. In this section, some aspects of efficiency that have guided the thinking that led to this report are reviewed. Increasing the energy efficiency of chips is always critical and has become more critical today with the competing desires for ‘greener’, cheaper components and for the ever-increasing size of arrays and storage required by A.I., big data, cryptography, and so on. The density of packaging of silicon chip components has also been steadily increasing for decades and this leads to an increase in the difficulty of an equal access path to different components on the chip. To improve this in modern CMOS technology requires increasingly complex geometries. Miniaturization of components increases energy losses: at the sub-micron scale and further down to the nanoscale, the resistance of the narrow, interfacial region encompassing the junction of the conventional conducting channel and the contact material (the latter also a conductor, but not necessarily of the same material) may dominate the resistance of the nanodevice, and therefore threaten to non-trivially impact the performance of mesoscopic or nanodevices. This is especially pronounced as the device size continues to shrink, and it could be worsened by the non-ideal nature of contacts themselves (such as vacancies, dislocations, impurities, surface roughness, etc.) [1,2,3,4,5]. Materials, regardless of their overall dimensions, can almost always be contacted on one or more of the surfaces, i.e., top, bottom, side. By modifying the shape of electrical contacts, in particular ones inspired by biologically inspired motifs, one could hypothesize that this may

a. Increase the effective contact area, without increasing power loss in the area of the contact;
b. Enable more efficient charge (and perhaps spin) transport via new transport pathways, and possibly through novel, hybrid modes of transport;
c. Allow examination of both material combinations and geometry combinations as tunable parameters.

This report focuses on the energy efficiency of transmitting signals to and from components via the contact (within the device/element) or through the interconnect (within the system) and uses inspiration from mechanical contacts in nature, specifically insect setae, the bristle-like structures that allow the insect to stick to surfaces, to design, fabricate, and test such bio-inspired contacts.

**Figure 1:**
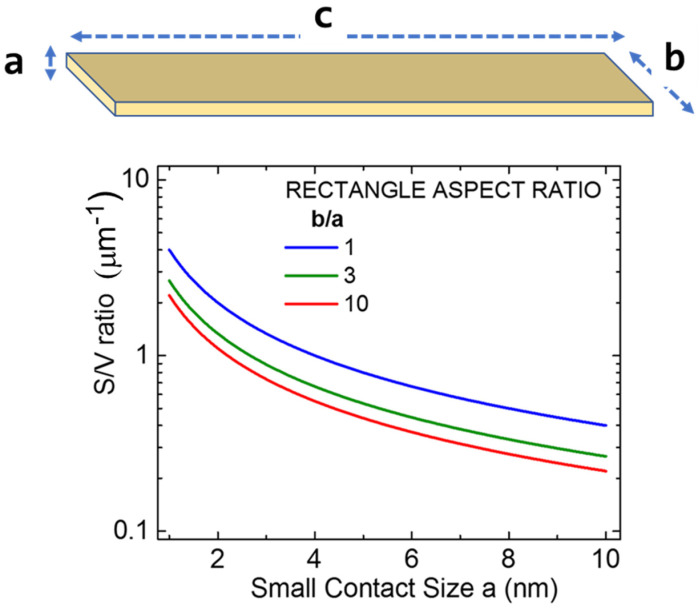
Surface-to-volume (S/V) ratio for anisotropic (*c* >> *b* > *a*) rectangular cuboid. The drawing illustrating the highly geometrically anisotropic rectangular meso-/nanowire is not to scale.

Ultimately, growth and nanofabrication on mesoscopic and nanoscales should involve considering the surface-to-volume (S/V) ratio. The larger the S/V ratio, the more states might be available for nanoengineering, and the more opportunities exist for utilizing the density of states (DoS) to benefit transport. The S/V ratio is shown in Figure 1 for a simple rectangular cuboid geometry, where the range of changes to this parameter as a function of the geometrical anisotropy is examined. Simply put, the S/V ratio is studied for the range of geometrical situations satisfying the inequality *c* >> *b* > *a*, while the characteristic length scale *a* is chosen as the smallest relevant growth and nanofabrication dimension. The S/V ratio is plotted as a function of length scale *a* in Figure 1, primarily because most of the nanofabrication techniques are either top-down or bottom-up processes, so that direction (“*a*” in the drawing) is often first subjected to the scaling efforts.

The study in Figure 1 shows that the *increase* in the size of the smallest length scale (say, thickness) *a* by one order of magnitude leads to the *decrease* in the S/V ratio value by approximately one order of magnitude. Additionally, one can observe that the S/V ratio is also influenced by the value of lateral anisotropy (here expressed as the *b*/*a* factor), but to a lesser extent. Now, modern nanofabrication techniques do not limit one to the use of a rectangular cross section (it would be represented by *a* × *b* here) of the highly elongated rectangular wire above, and one can examine other cross sections (circle, ellipse, triangle, etc.), but the main changes to the graph shown in Figure 1 end up being quantitative in nature.

It should be noted that the plot in Figure 1 is scalable across different ranges, so the choice of technologically relevant dimension (thickness *a*) is the only explicit input, as long as the rectangular shape is highly anisotropic, as indicated.

Transmitting signals accurately requires consideration of the noise in the channel, and this area of research has been underrepresented for nanowires. When it has been investigated, it has been often done so for nanowire transistors [6,7,8], but also for gas sensors [9,10], UV or light sensors [11,12,13], and photodiodes [14,15]. There are several spectrally differentiated types of noise, generally found in nanowire devices, but the main thrust of experiments and analysis focuses on 1/*f* or pink noise, and 1/*f* ^2^ or Lorenzian noise.

Hooge [16] found a simple formula for experimental low-frequency noise which relates the number of carriers, square of the current, and frequency to the spectral density and a material-dependent Hooge parameter, $\alpha_{H}$, which was initially thought to be a universal constant with the value $2\times 10^{-3}$. In bulk silicon, the Hooge parameter is around $10^{-5}$ [17], and for the nanowire devices that have been measured, the Hooge parameter varies from this value to larger ones $10^{-3}$ to $10^{-2}$; as the Hooge parameter is related to how many traps there are trapping and detrapping carriers (a cause of current noise), these high values imply that this effect is serious [17].

Despite it being generally unwanted, noise can have uses. Recording low-frequency noise could be used to probe trapping defects in devices [18]. Noise spectroscopy based on 1/*f* noise can give information such as the volume of traps in the gate dielectric; analysis of Lorenzian noise gives individual trap parameters [19] and is a useful technique for improving device fabrication and design. For example, it allows the designers to discover that traps have been caused in their device by the unintentional incorporation of catalyst (Ni) in VLS-grown Si NWs [20], while an interesting paper [21] presented a way to measure InAs nanowire MOSFETs with either the inner core channel or outer surface channel dominating the transport, and this allowed the measurement of Hooge parameters for the material without the surface effect [21]. A tunable noise suppressor has been created, where a magnetic field can change Ni nanowire resonant frequency, which changes the frequency range of noise suppressed [22]; finally, noise can remove chaos from a system [23]. In general, one wants to reduce the noise, especially the surface and contact noise, in order to have reliable devices as device sizes shrink to the nanoscale.

Additionally, noise levels give a limit to the detectability of sensors [23,24,25,26], as the constant trapping-detrapping at the nanowire surface gives rise to a background signal, as in UV sensors [11].

There are at least two main causes of low-frequency noise: mobility fluctuations (see, for example, [27]) and number of carrier fluctuations (see, for example, [28]). Lorentzian noise is caused by thermal activation of traps at a single energy level or tunneling from trap to trap at certain spatial locations. To manifest 1/*f* noise, the carriers follow a random walk along nanowire surface states, and an analytical theory relating the Hooge parameter to temperature, carrier concentration, radius, and surface density of states in the nanowire has been proposed [17]. Experimental evidence for such a mechanism comes from the effect of passivation of the surface on noise (e.g., [11]) although it did not decrease the noise in [29] (but was found to reduce temperature-related energy loss). Temperature also plays an important role, as shown, for example, in InAs nanowire transistors, which were found to have 1/*f* noise except at low temperature where thermally activated Lorenzian noise was seen [30]. Similarly, Si nanowire FET showed correlated mobility fluctuations at high temp (300 K) and low temp (100 K); the noise is best modeled by the carrier fluctuation model [31]. Frequency can have an effect, too—junctionless Si nanowire transistors demonstrate different types of noise over different frequency ranges [32]. Thus, noise can also be reduced by changing the way the device is operated. An interesting approach is to apply a d.c. bias larger than the height of the Schottky barrier, to have an excitation independent from the a.c. excitation: this reduces the noise and, because it changes it from 1/*f* ^2^ to 1/*f*, implies that this approach drastically reduces contact noise leaving the 1/*f* nanowire noise [33]. In InAs nanowire transistors, it was found that contact noise dominates the resistance and noise profile when the device is fully on; near or below the threshold, the channel dominates both resistance and noise [34].

Deviation from 1/*f* noise in Si nanoscale MOSFET is also possible, as illustrated by a model of one-by-one trap activation controlled by gating voltage. In this case, Coulomb repulsion between adjacent charged trap sites causes reduced noise by an order of magnitude; the effect decreased if the electron density was high enough to screen the charges [35].

More critical to the research results reported in this paper, the existence of noise mechanisms related to the surface area of a device and relative size of the contacts strongly implies that the shape, length, width, and geometry of devices will have an effect on noise levels. For example, in SiN_x_ insulator-gate GaAs nanowire FETs, the drain current noise increases with nanowire width and decreasing gate length, and the noise changes from 1/*f* to 1/*f ^2^* as the device size decreases (although this is not a general result as this is not observed with Schottky-gate nanowire FETs) [36]. From theoretical analysis, the authors found that traps with short time constants affect the slope of the spectrum, while those with long time constants shift the spectrum, and thus concluded that the change in noise type is due to the broadening of the time constant distribution [36].

Thinner nanowires move traps away from the interface, reducing noise. At high drain currents, Si nanowire MOSFETs demonstrate noise spectral density decreasing rapidly with drain current (caused by ultranarrow source/drain extension regions of the Si nanowire transistor) [37]. A 3D (Si nanowire) transistor was found to outperform a 2D (planar) transistor [38] because, as is shown by simulation, decreasing nanowire diameter reduces trap density and moves traps away from the interface [39].

If contact noise has a big effect, an obvious solution is to suggest parallelization of nanowires [8,40] as this will reduce the relative amount of contact noise whilst still keeping any gains from shrinking the device dimensions. It has been found that silicon-oxide-nitride-oxide-silicon (SONOS) devices with multiple nanowires outperform standard devices as small nanowires have fewer grain boundaries comparable to grain size [40]. However, if subjected to the Fowler–Nordheim tunneling program/erase operation, the difference between multiple nanowires and the standard single channel was decreased due to the introduction of traps [41]. Even worse, the number of oxide traps that can be generated by Fowler–Nordheim tunneling stress was found to be higher in cylindrical (NW) compared to 2D (planar) FinFETs and is due to an increased electric field at the SiO_2_ interface [42]. These results suggest that noise spectroscopy before and after FN stress should be a standard characterization test for such devices and that it might be fruitful to look for ways to operate the devices that reduces the amount of FN tunneling if one wishes to reduce contact noise from nanowire transistor devices.

To conclude, thanks to this relatively broad but focused review, it is partially deduced and partially intuited that a more radical change in the shape of contacts is worth examining, which leads to the next section, where elaboration on bio-inspired design is provided in more detail.

## 2. Inspiration from Nature

Nature offers numerous sources for inspiration [43] and generic blueprints for tried and tested approaches to solve complex mechanical [44] and even computational problems [45,46]. Biological micro- and nanotribology [44] has been studied extensively, in part because of the commonalities between biological and technical microsystems: in both systems, interaction occurs on roughly the same force and length scales, and surface properties such as wettability have a strong impact on system performance [44,46,47]. While in technical microsystems mechanical properties such as friction and adhesion are determined almost exclusively by the topmost layer, biological systems are far more complex due to the influence of the entire biological object through properties such as viscoelastic constitution [44].

Contact formation for substrates depends on the ability to adapt to the different surface topographies of the substrate [48]. The state of the art of biological sensors and bio-inspired technologies [49] currently allows researchers to measure properties of biological structures such as adhesion [43], stress, and friction [47], and to do so on micro- and nanoscales [44]. This opens the door for the use of such structures [46] when engineering technical or mechanical microsystems [50,51], as well as electrical systems [52,53,54], specifically to design more efficient electronic contacts.

Insects, besides being of utmost ecological significance, are known to be extremely diverse and can feature highly evolved sensory systems, shaped by millions of years of adaptation [50]. In this article, a biologically inspired electrical contact design using insights into the approximated shape of insect setae is investigated. These hair-like microscale structures are used to generate friction (for movement) and adhesion (to stay in place), as well as for tactile sensing, and have been shown to exhibit remarkable adaptability and contact efficiency.

## 3. Materials and Methods

### 3.1. Simplified Shape of Biological Contacts

**Figure 2:**
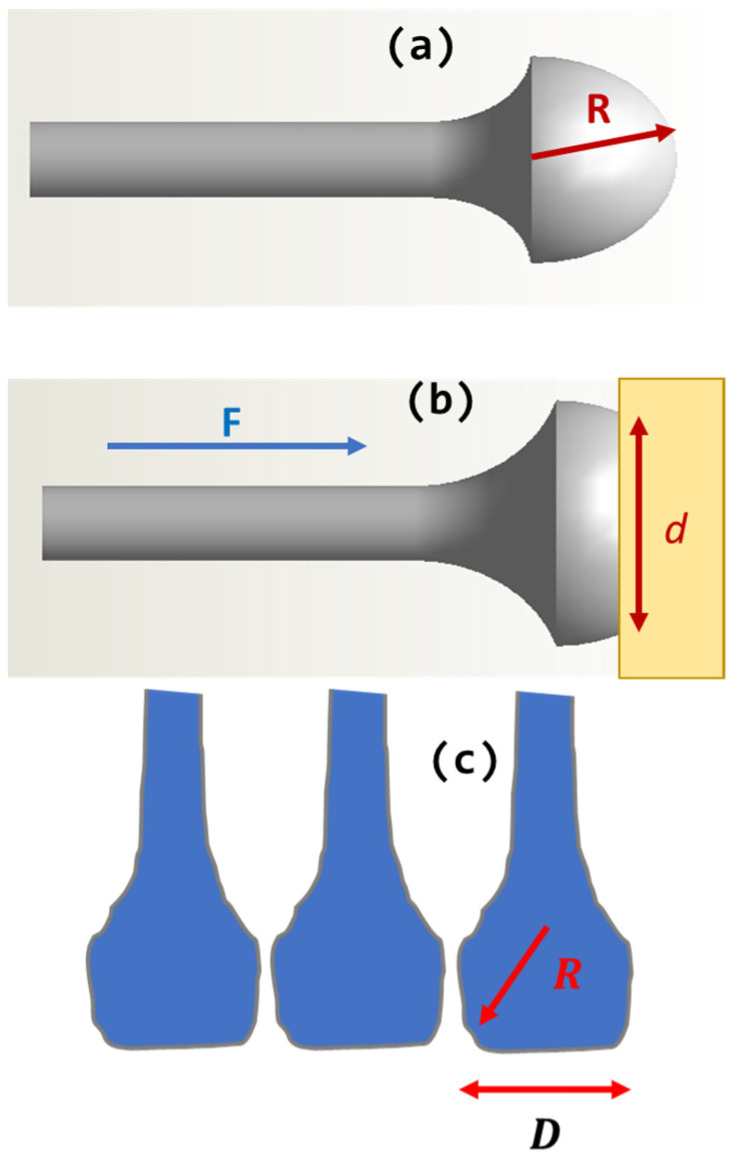
A design option in mimicking insect setae contacting an object from an external world, in this case a rectangular yellow block. (**a**) a simplified model of setae; (**b**) a modification of setae in contact with the outside world with some defined force ***F***, and occurring across a reasonably defined laterally extended *d*; (**c**) a series of parallel shapes, modeled after a modified tip of setae, with two characteristic length scales.

As one may surmise from the introduction and the section on drawing inspiration from nature, two main lines of research and development work influenced the design, materials, and methodology overall. In one case, this report follows the findings from the last two decades, where Arzt et al. [50] showed how mechanical properties of biological contacts offer useful lessons for the overall design, while Gao et al. [51] point to a curious and very motivating fact that some materials with imperfections may not have their properties negatively affected by those imperfections once the nanoscale is reached. Figure 2 below shows a simplified form of a single element mimicking insect setae that is implemented and analyzed in this report.

Two contact shapes were designed, following a variety of options of contact shapes available in the biological world. It should be noted that the report lays no claims that either of the shapes is the best by some performance measure over a variety of other possible bio-inspired shapes of electrical contacts.

### 3.2. An Overview of the Project Flow

**Figure 3:**
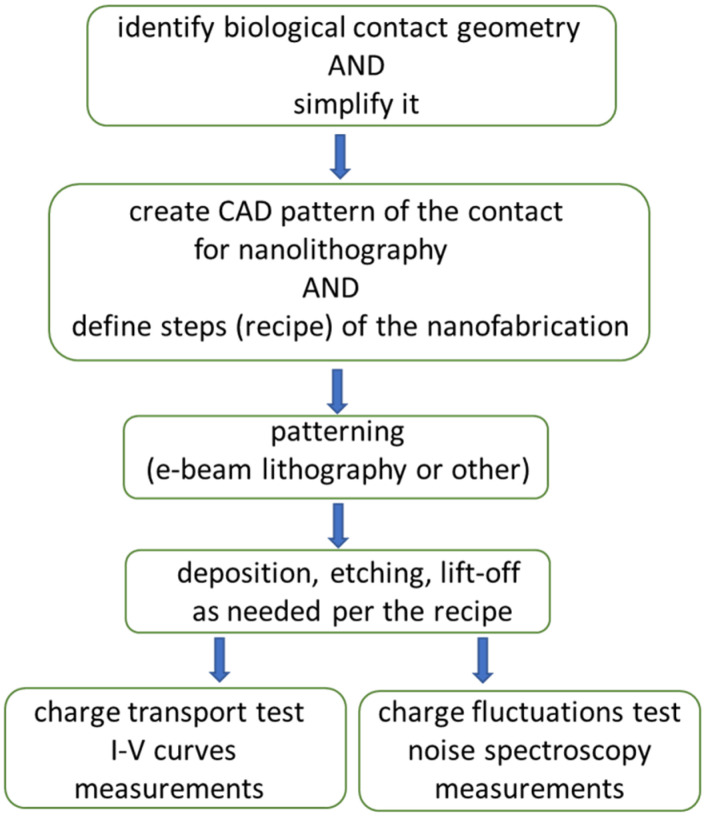
Flowchart displays steps undertaken from the initial idea to the testing results reported.

Given the somewhat unusual nature of the project, a flowchart depicts how the research and development work proceeded for the work reported here. Figure 3 shows the main phases of the work.

### 3.3. Setting up the Pattern for Testing

Two contact shapes were developed, in addition to the rectangular, control shape. In all cases, the voltage probes are in the form of a standard, bottom-up pattern, labeled (V_i_^+^, V_i_^−^), where *i* = {1, 2…}, often used in testing of nanodevices. In contrast with that design choice, the current contacts have one side that is a standard rectangular side contact, while the other side is the choice of bio-inspired contact. The current contacts are labeled (I*_j_*^+^, I*_j_*^−^), where *j* = {a, …, d}.

**Figure 4:**
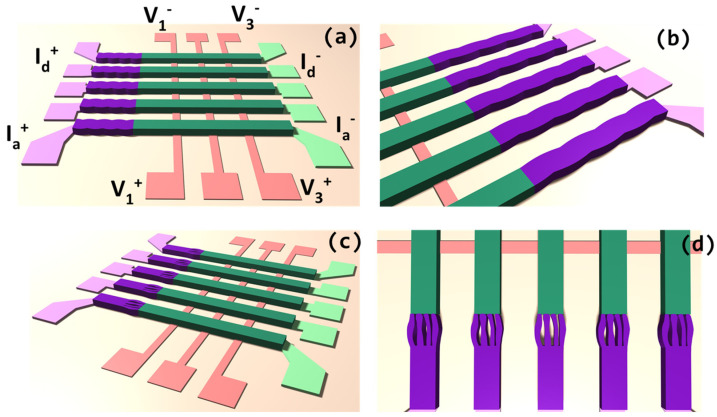
An overview of the full pattern test nanofabrication design is shown in panels (**a**,**c**). The detailed look focusing on the contact shapes examined in this report is presented in panel (**b**) for wave-like, and in panel (**d**) for finger-like contacts. The color scheme is meant to be a guide for the definition of the elements, so pink is reserved for voltage probes, the light green is the side probe pad for current contact, and the light magenta is the probe pad for the novel current contacts made from NiSi_2_, themselves being presented in dark magenta color. Lastly, the dark green represents the actual Si mesowire.

Figure 4 below shows the patterns and the testing configuration. Two shapes are chosen for nanofabrication and testing. One is called “finger-like”, reminiscent of the shape of fingers in Figure 2c, and the other “wave-like”. The finger-like contacts are actually a simplified form of insect setae, as shown in Figure 2 above. The wave-like pattern is meant to be tested as a crossover between standard rectangular shapes and a non-trivially curved finger-like, setae-inspired contact, essentially to see if simple geometry has an influence on a scale different than the bio-inspired contact.

## 4. Results and Discussion

### 4.1. Electron Microscopy Images of the Nanofabricated Bio-Inspired Contacts and Testing Setup

**Figure 5:**
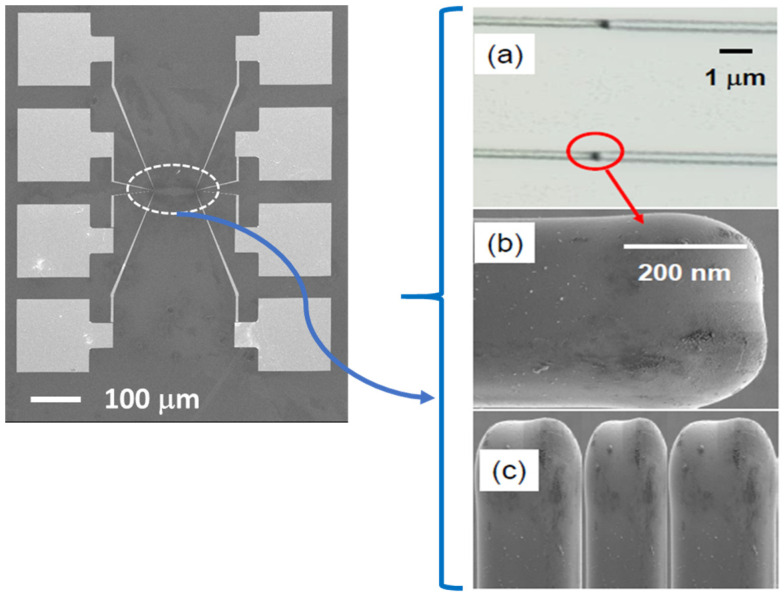
(**left**) A large-scale scanning electron microscope (SEM) image, showing the contact pads where one connects the outside instruments, such as current source and voltmeter, together with the small micrometer-scale wiring that leads to the sub-micron details shown on the right. A dotted white oval represents the detailed features of the nanofabricated mesowires and nanocontacts on the right; (**right**) SEM images of the details of nanocontact. (**a**) the contact to mesowire is shown; (**b**) a single contact in the finger-like shape is shown; (**c**) a series of finger-like contacts next to each other, as intended by the design shown in Figure 4d.

The patterns outlined in Figure 4 were transferd to the wafer using e-beam lithography, lift-off, and deep reactive ion etching, while the large area (~100 μm) probe contact pads were fabricated using optical lithography, together again with etching and lift-off. The substrates are commercially available wafers of silicon (Si), on top of which NiSi_2_ film is deposited, and where the new contacts are then made (wavy, finger-like) as well as standard rectangular contacts. Mesoscale wires are made from Si. The standard contacts on the opposite side of the bio-inspired contacts are made from titanium and gold (Ti + Au). Figure 5 shows the large area on-chip testing configuration on the left, where one connects the on-chip contact test system with the outside world, such as current source, voltmeter, etc. On the right of Figure 5, the three panels are visible, labeled (a), (b), and (c), representing different scales of the bio-inspired contact, all parts of the small detail shown in the dotted oval in the center of the panel on the left of Figure 5. Panel (a) of Figure 4 shows the “bird’s eye view” image of the mesowire and the contact joint.

The current-voltage, I–V, measurements were performed using the Keithley*^®^* SCS 4200 system, with the wafer carrying the patterned features from Figure 5 to establish the electrical connections from the contacted nanowires to the instrument. Two groups of experiments were performed. In the first group, the I–V curves are measured for three types of contacts at room temperature, motivated by the room temperature use of most on-chip consumer electronics. In the second group of measurements, the small wafer with the nanofabricated contacts and nanowires is placed into the closed cycle cryostat, and the I–V measurements are performed at a fixed, lower temperature.

### 4.2. Transport Characteristics of the Bio-Inspired Contacts

**Figure 6:**
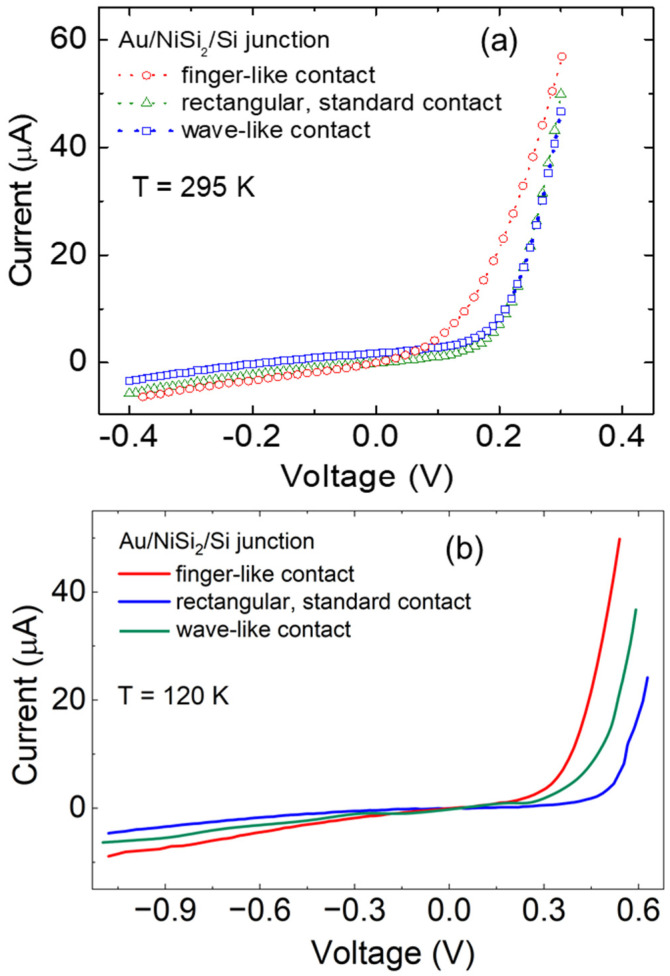
(**a**) Room temperature I–V curves for three contact geometries: standard rectangular (green), insect setae-motivated, finger-like (red), and wave-like (blue). (**b**) Low-temperature (T = 120 K) I–V curves for the same contact geometries. Same color scheme applies as in panel (**a**). Note that both voltage and current scale change with temperature.

Figure 6 below shows a comparison of the outcomes of the two groups of measurements. In Figure 6a, the outcome of room temperature measurements is shown. Standard rectangular contact and the wave-like contact differ from each other by small amounts in their (V_i_, I_i_) values, as seen in the near overlap of their values in Figure 6a. In contrast, the insect setae-motivated, finger-like contact differs from both in a measurable way, and it has a “turn ON” point at a lower voltage, when compared to the same current value. This result is the first indicator that the hypothesis explored might be valid. Figure 6b displays additional interesting results. Due to the lower temperature, the voltage scale is understandably broader, but more interesting is that the three contacts are now clearly differentiated from each other. The finger-like contact still shows the best performance, as measured by the lower “turn ON” voltage value at fixed currents. There is now a clear, quantitative distinction between the standard rectangular contact and the wave-like contact. Interestingly, the wave-like contact performance falls in between the finger-like and the rectangular one, roughly. This points towards the importance of the scale choice in the geometry of the wave-like contact, which is similar at the contact point for finger-like and wave-like, but also points to the importance of the actual end shape being different between the finger-like and wave-like contacts.

### 4.3. Fluctuation and Long-Term Stability

**Figure 7:**
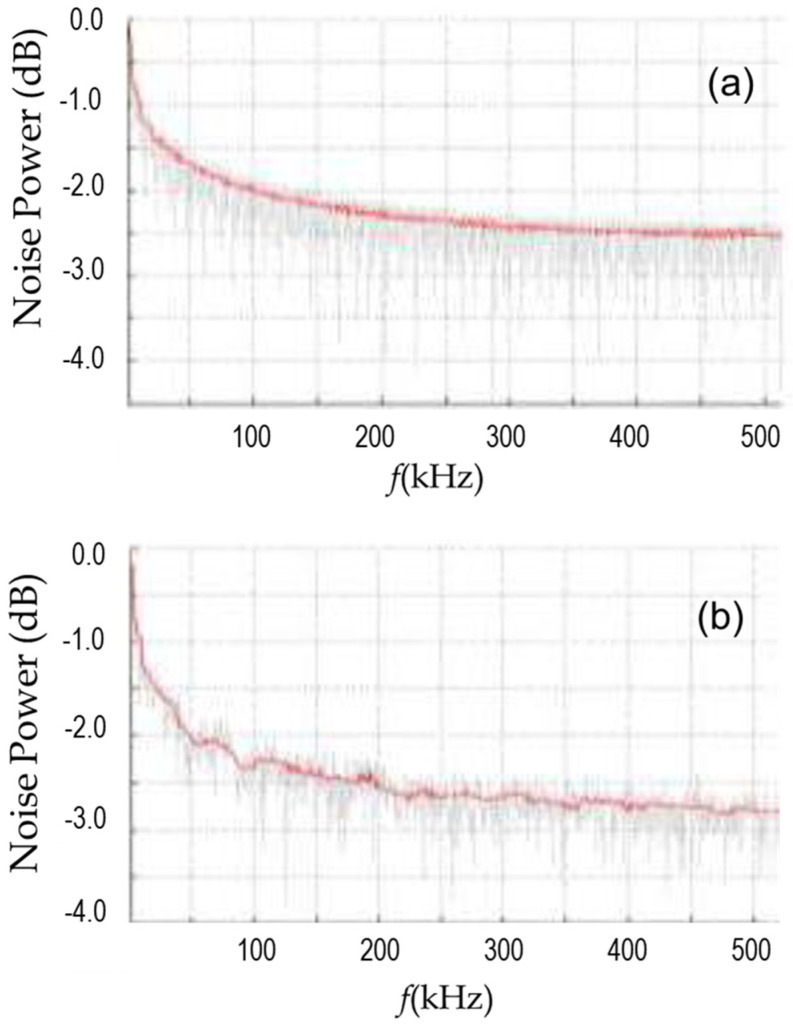
(**a**) Broadband noise spectrum for a mesowire with the standard, rectangular nanocontact at room temperature. (**b**) Broadband noise spectrum for mesowire with the bio-inspired, finger-like nanocontact at room temperature. Both plots have 30 Hz frequency resolution.

Given the strongly suspected role of geometry, it seemed prudent to measure broadband 1/*f* noise spectra. In a vast number of noise studies, some version of the Hooge [55] relationship applies:

(1) $$\frac{S_{I}\left(f\right)}{I^{2}}=\frac{S_{R}\left(f\right)}{R^{2}}=\frac{\alpha_{H}}{fN}$$

where *I* is the current, *f* is the frequency, *N* is the number of carriers, and $\alpha_{H}$ is the Hooge constant. A closer look at the noise spectra in Figure 7 demonstrates the challenges in using the Hooge equation. Namely, unlike the standard contact, for the bio-inspired contacts, the anticipated linear “fit” of the power vs. inverse frequency on a semi-logarithmic scale does not describe the system as well. This is evident from the raw data, which are shown here, instead of processed semi-log fit, because one can see the non-trivial changes in the strength of the signal as a function of the frequency, unlike in the case of data for a standard, rectagular contact. The causes of such behavior are a subject of an ongoing investigation, and the following are some hypothetical explanations suggested:

(a) It is known that the contributions to Equation 1 above originate from two main sources, the contact interface and the conduction channels. It would appear that two mechanisms’ contribution changes several times during the frequency sweep in the range [0, 500 kHz];
(b) The consideration in the hypothesis (a) notwithstanding, it will be important to examine whether the Hooge constant *α*_H_ is actually a constant under the conditions of the experiment here.

Naturally, one cannot completely eliminate the possibility that the contact inhomogeneity is still present, but at the scale at which the tests were performed so far, no evidence of it has been found. Future work will be needed to gain insight about more saddle noise mechanisms. For example, it is evident that the noise power is overall lower for the bio-inspired contact in Figure 7, but there is not a definitive explanation for why the curve shows more wiggles (mini-peaks).

## 5. Conclusions and Future Work

Several main conclusions are listed below, followed by a discussion on some open questions and likely future work.

1. It is shown that it is possible to design and nanofabricate biologically inspired, insect setae-based nanoscale contacts.
2. Two different non-standard nanocontact shapes for meso- and nanowires were designed and nanofabricated, finger-like protrusions based on insect setae in parallel and a wavy interface seen as a hybrid of this structure and the standard rectangular contacts.
3. Charge transport measurements, in the form of I–V curves at room and low temperatures, show how the identical chemical interface (NiSi_2_-to-Si) of nanocontacts to mesowires leads to variations in (*V*_i_, *I*_i_) data, thereby confirming the hypothesis that the energy (power) for charge transport through a junction may be modified by the shape of the contact.
4. The results show that, at room temperature, the setae-like contacts offered the best performance in terms of turn-on voltage, and, intriguingly, also offered lower noise power than the rectangular contacts across the whole range of frequencies with the concomitant oddity that this noise spectra were not fit by the Hooge equation as good. Thus, this work suggests that the setae-inspired contact is a good new design for standard meso- and nanoelectronics.

One may ask the question as to what part of the design achieves these improvements over standard rectangular contacts, the curved interface or the narrow finger-like shapes behind them. As the wavy contact I–V curves are much closer to the rectangular contacts at room temperature, this suggests that the finger-like aspect of the design is the most important. The results from the low-temperature studies demonstrate that the curved contacts do have an effect as the wavy contacts are a significant improvement over the rectangular contacts, but this effect is lessened at higher temperatures. As noise is reduced in thinner wires due to the traps being moved away from the interface, this may explain the reduction in noise power for the setae-like contacts. It is possible that the difference in resonant frequencies due to either or both the curved interface (offered by both the wavy and setae designs) and the narrowness of the contact design may be the cause of the dips in the noise spectrum that move the experiment away from the Hooge theory.

The report makes no claims that either of the shapes tested so far is the best by some performance measure over a variety of other possible bio-inspired shapes of electrical contacts, and it is clear that continued investigation is warranted, for other shapes, length scales, and material combinations of interest to nanoelectronics.

Ongoing and future work includes exploring modifications of the initial design and attempting to explain how data for three different contact shapes depend on the shape, aspect ratio, and related parameters using techniques such as nanoscale thermal imaging and spatially resolved surface probe microscopy, among others. For example, it is clear that the temperature-dependent data [56] needs to be taken with various techniques (when appropriate and feasible), due to a reasonable hypothesis that, at these length scales, electron scattering becomes modified by the shape of the boundary of the conducting channel. In parallel, a computational effort will be needed to elucidate the nature of transport and scattering when channels have such shapes. In addition to a reasonable working hypothesis that energy efficiency differences stem from power considerations (i.e., the product of current and voltage at different geometries), a large-scale computational study accounting for the contact shape is likely to provide more clarity about the impact of various geometrical and material parameters. Finally, to create the best contacts, it will also be worth exploring how different material combinations interact with shape to produce different properties.

Among the significant outcomes of this work are the following:

(a) Experiment-based finding that there are bio-inspired methods for increasing the energy efficiency of switching.
(b) Transport and broadband noise data correlate as indicators of efficiency.

Among the limitations, it is important to keep in mind there are technical and logistical limitations to nanopatterning of bio-inspired features. Since all patterns in e-beam lithography are the result of some “matrix pixelation” representation, representing a curvy shape on the 200 nm scale is different from representing the same shape on the 20 nm scale, in terms of time and energy for, and an error rate of patterning. Practically, this means that there are limits to which shapes and patterns can be effectively represented and repeatedly scaled up. Future advances in nanofabrication techniques might address this issue, as it appears inevitable some bio-inspired designs will be of interest to industrial-scale nanofabrication.

## Data Availability

Data are available upon reasonable request from the corresponding author.
